# Loss of p53 enhances the function of the endoplasmic reticulum through activation of the IRE1α/XBP1 pathway

**DOI:** 10.18632/oncotarget.4598

**Published:** 2015-06-23

**Authors:** Takushi Namba, Kiki Chu, Rika Kodama, Sanguine Byun, Kyoung Wan Yoon, Masatsugu Hiraki, Anna Mandinova, Sam W. Lee

**Affiliations:** ^1^ Science Research Center, Kochi University, Kohasu Oko-cho Nankoku-shi, Kochi, Japan; ^2^ Cutaneous Biology Research Center, Massachusetts General Hospital and Harvard Medical School, Charlestown, MA, USA

**Keywords:** tumor suppressor p53, p53 target genes, ER function, IRE1α/XBP1 pathway

## Abstract

Altered regulation of ER stress response has been implicated in a variety of human diseases, such as cancer and metabolic diseases. Excessive ER function contributes to malignant phenotypes, such as chemoresistance and metastasis. Here we report that the tumor suppressor p53 regulates ER function in response to stress. We found that loss of p53 function activates the IRE1α/XBP1 pathway to enhance protein folding and secretion through upregulation of IRE1α and subsequent activation of its target XBP1. We also show that wild-type p53 interacts with synoviolin (SYVN1)/HRD1/DER3, a transmembrane E3 ubiquitin ligase localized to ER during ER stress and removes unfolded proteins by reversing transport to the cytosol from the ER, and its interaction stimulates IRE1α degradation. Moreover, IRE1α inhibitor suppressed protein secretion, induced cell death in p53-deficient cells, and strongly suppressed the formation of tumors by p53-deficient human tumor cells *in vivo* compared with those that expressed wild-type p53. Therefore, our data imply that the IRE1α/XBP1 pathway serves as a target for therapy of chemoresistant tumors that express mutant p53.

## INTRODUCTION

Cancer cells induce distinct alterations of metabolic pathways, including glycolysis and protein synthesis, to survive and proliferate under conditions of stress associated with tumor growth such as nutrient limitation and anaerobic stress [1–3]. Cancer cells also synthesize a large amount of protein to support their rapid growth [4]. The endoplasmic reticulum (ER) is a specialized intracellular organelle responsible for the proper localization, modification, and folding of proteins. Metabolic and anaerobic stress induce ER dysfunction and the unfolded protein response (UPR). UPR maintains and restores ER homeostasis by increasing protein secretion through induction of ER chaperons that mediate protein refolding and by degrading unfolded proteins. However, irreversible ER stress induces cell death to eliminate damaged cells. Thus, increasing the function of ER and its resistance to ER stress is essential for tumor proliferation and survival, and these processes are implicated in the enhancement of ER function in diverse types of human cancer cells [5, 6].

Three types of ER transmembrane proteins, protein-kinase/endoribonuclease inositol-requiring enzyme 1 alpha (IRE1α), protein kinase R-like ER kinase/pancreatic eIF2 kinase (PERK), and activating transcription factor 6 (ATF6) mediate the mammalian ER stress response as well as UPR [5]. IRE1α is a key signal transducer that maintains ER function. The IRE1α signaling pathway induces expression of the transcription factor XBP1(S), which is the active form of XBP1 generated by IRE1α-dependent splicing of *XBP1* mRNA. XBP1(S) increases the expression of ER chaperons and ER mass, stimulates lipid biogenesis, and degrades unfolded proteins to enhance the secretory function of ER and to suppress ER stress-mediated cell death [7–9]. In particular, gain of secretory function of ER stimulates the production of growth factors such as VEGF [10, 11]. Moreover, the activated IRE1α/XBP1 pathway plays an essential role in resistance and adaptation to ER stress by many types of cancer cells [2, 6, 12]. However, the specific regulatory mechanism of activation of the IRE1α/XBP1 pathway in cancer cells is unknown.

The tumor suppressor p53 gene is mutated in at least one-half of human cancers, and defects in the p53 response pathway promote tumor development [13]. The functions of p53 influence the cell cycle, DNA repair, apoptosis, and nuclear vesicular trafficking in response to cellular stress such as DNA damage, oncogene activation, and hypoxia; however, the role of p53 in ER function is largely unknown [14, 15].

Here we demonstrate that p53 acts as an important regulator of ER function via suppression of the activation of the IRE1α/XBP1 pathway. Upon ER stress and homeostatic conditions, the splicing of *XBP1* mRNA and the levels of XBP1(S) are stimulated in p53-deficient cells. Here we show that loss of p53 function induced IRE1α expression by inhibiting the p53-dependent association of IRE1α with synoviolin-1 (SYVN1) which induces degradation. Moreover, an IRE1α inhibitor STF-083010 suppressed protein secretion, induction of cell death, and tumor growth *in vivo* in p53-deficient human tumor cells but not in those that expressed wild-type p53. Our findings reveal a novel mechanism for the regulation of IRE1α expression by p53. Thus, the regulation of the IRE1α/XBP1 pathway by the p53–SYVN1–IRE1α complex represents a new mechanism for increasing ER function in cancer cells.

## RESULTS

### Loss of p53 function activates the IRE1α/XBP1 pathway

**Figure 1:**
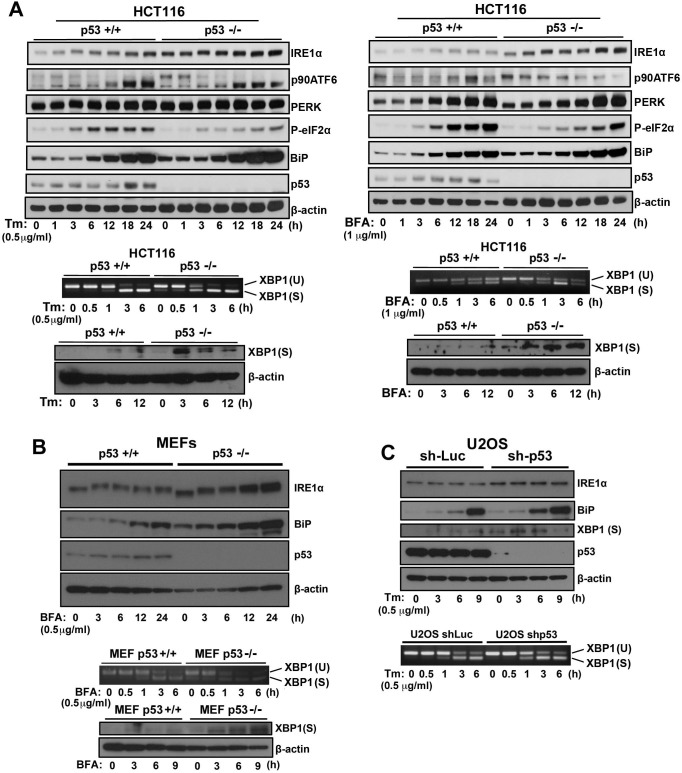
ER stress response in p53-deficient or knockdown cells **A.** HCT116 *p53*^+/+^ or HCT116 *p53*^−/−^ cells, **B.** MEF *p53*^+/+^ or MEF *p53*^−/−^ cells, and **C**. U2OS shLuc or U2OS shp53 cells were incubated with Tm (0.5 μg/mL) or BFA (1 μg/mL) for the times indicated. Cell lysates were analyzed using western blotting with the indicated antibodies. The blot was cut based on the size of proteins or stripped. Total RNAs were extracted and subjected to RT-PCR analysis using specific primer sets for XBP1(U) and XBP1(S). Cell lysates were analyzed using western blotting with indicated antibodies.

To understand the role of p53 in the ER stress response mediated by the IRE1α/XBP1, ATF6, and PERK/eIF2α signaling pathways, we treated HCT116 *p53^+/+^* and HCT116 *p53*^−/−^ cells (Figure 1A), MEF *p53*^+/+^ and MEF *p53*^−/−^ cells (Figure 1B), and U2OS-shLuc and U2OS-shp53 cells (Figure 1C) with inducers of ER stress, brefeldin A (BFA), tunicamycin (Tm), or both, to determine the expression of proteins that mediate the ER stress response. p53 deficiency obviously affected IRE1α expression level compare to p90ATF6 cleavage (decreasing p90ATF6 expression) and phosphorylation of eIF2α by PERK upon ER stress (Figure 1A). Depletion or knockdown of p53 expression increased IRE1α and BiP expression in the absence of BFA and Tm treatments; furthermore, p53 deficiency enhanced the induction of IRE1α and BiP expression upon ER stress. During ER stress, active IRE1α splices *XBP1* mRNA to generate *XBP1(S)* mRNA that encodes an active form of XBP1, XBP1(S), which initiates a major UPR program including the induction of ER chaperons such as BiP.[5] Therefore, we investigated whether the induction of IREα upon ER stress translated to downstream activation of XBP1 in p53-deficient cell lines. Consistently, we observed enhanced *XBP1* mRNA splicing and induction of XBP1(S) protein expression in p53-deficient cells in response to ER stress. Notably, basal IRE1α protein and spliced XBP1 mRNA levels were moderately elevated in the absence of ER stress agents, suggesting that not only does loss of p53 function potentiates the IRE1α/XBP1 pathway of the UPR upon ER stress but p53 function may have an inhibitory effect on the pathway. Thus, increased BiP expression in p53-deficient cells was induced by increased XBP1(S) expression. These results suggest that p53 regulates IRE1α expression, and loss of p53 function induces IRE1α expression and activation of the IRE1α pathway, stimulation of *XBP1* mRNA splicing, and XBP1(S) expression in the presence and absence of ER stress.

### IRE1α expression is regulated by wild-type p53 function

**Figure 2:**
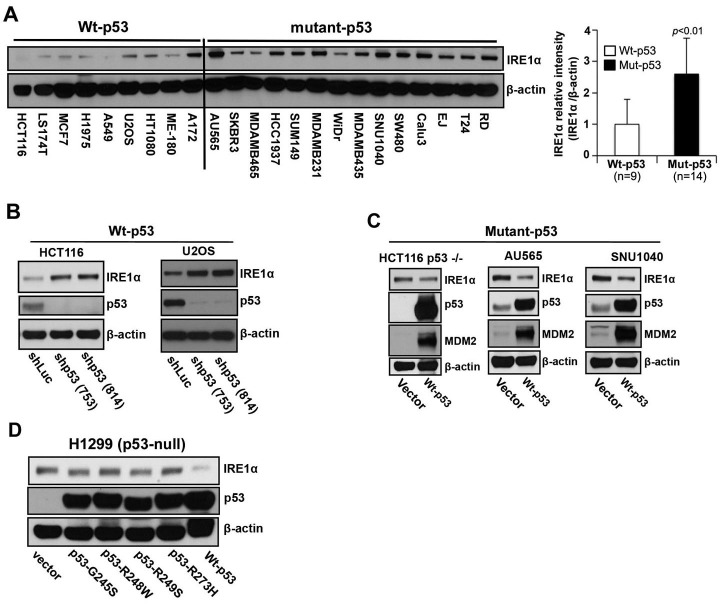
IRE1α expression is regulated by p53 **A.** Western blot analysis of the expression of endogenous IRE1α in 23 human cancer cell lines. Cell lines were grouped according to expression of wild-type or mutant p53 as indicated. (A well between wt-p53 and mutant-p53 cell lines was cut, from the gel as indicated by a black line, due to the controversial p53 status of the cell line). Right panel: The intensities of the IRE1α bands (left panel) are expressed relative to those of β-actin. Values shown are the mean ± standard deviation (s.d.). The *P* value was calculated using two-way ANOVA. **B.** Downregulation of p53 expression induces increased expression of IRE1α. HCT116 *p53*^+/+^ and U2OS cells were transfected with shLuc, shp53 (753), or shp53 (814), and selected using puromycin. Whole cell lysates of a pool of transfectants were analyzed using western blotting with the indicated antibodies. **C.** Overexpression of wild-type p53 inhibits IRE1α expression in mutant-p53 cell lines. Cell lysates, prepared 48 h after transfection with wild-type p53, were analyzed for the expression of indicated proteins. **D.** Mutant p53 proteins do not inhibit IRE1α expression. Cell lysates were prepared from cells transfected with p53-G245S, p53-R248W, p53-249S, and p53-R273H expression vectors or from cells that constitutively expressed wild-type p53 and were analyzed for the expression of the indicated proteins.

To support our hypothesis that loss of p53 function derepresses IRE1α expression, we analyzed nine wild-type p53- and 14 mutant p53-expressing human cancer cell lines to determine whether endogenous IRE1α expression levels were affected by p53 status. Western blot analysis showed that IRE1α was abundantly expressed in 12 out of 14 cells lines that expressed mutant p53: AU565, SK-BR-3, HCC1937, SUM149, MDAMB231, MDAMB435, SNU1040, SW480, Calu3, EJ, T24, and RD (Figure 2A). In contrast, the expression levels of IRE1α were significantly lower in cells that expressed wild-type p53. To corroborate these findings, we either knocked down p53 expression in wild-type p53 cells or overexpressed wild-type p53 in mutant p53 cells and measured IRE1α expression levels. Stable knockdown of p53 (shp53-753 and shp53-814) in HCT116 *p53*^+/+^ and U2OS cells increased the levels of IRE1α compared with that of control cells (shLuc) (Figure 2B). Similarly, transient expression of wild-type p53 in HCT116 *p53*^−/−^, AU565, and SNU1040 cells decreased IRE1α expression (Figure 2B). Furthermore, transient expression of several mutant p53 forms (p53-G245S, p53-R248W, p53-R249S, and p53-R273H), which lack DNA-binding and transactivation function, in p53-null H1299 human cancer cells had no effect on IRE1α expression; contrarily, only expression of wild-type p53 reduced IRE1α expression (Figure 2C). This suggests that wild-type p53 function negatively regulates IRE1α expression.

### p53 stimulates the degradation of IRE1α by the proteasome

**Figure 3:**
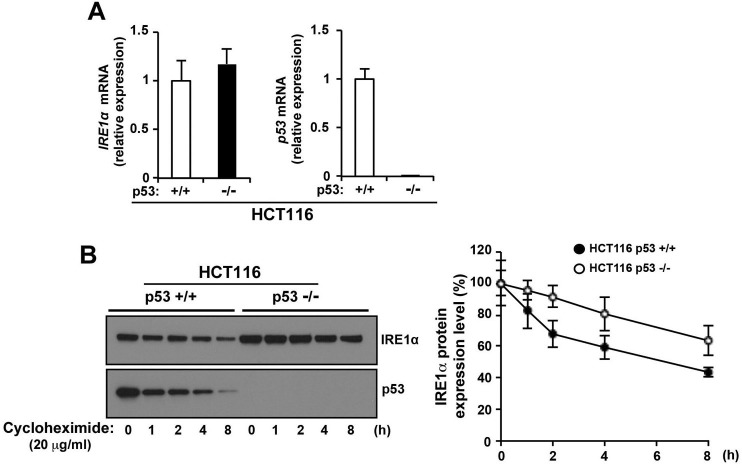
p53 stimulates IRE1α protein degradation **A.** The level of p53 did not affect the expression of *IRE1α* mRNA. Total RNAs were extracted and subjected to qRT-PCR analysis using specific primer sets for *IRE1α*, *p53*, and *GAPDH*, and the data were normalized to those of *GAPDH*. Data shown are the mean ± s.d. (triplicates measured at the same time). **B.** p53 promotes IRE1α degradation. The indicated cells were treated with cycloheximide (25 μg/mL) for 1 h and incubated further for the indicated times. Cell lysates were analyzed for the expression of the indicated proteins. The blot was cut based on the size of the proteins of interest (left panel). The intensities of the IRE1α bands were determined (one of the gels is shown in left panel) and are expressed relative to those of β-actin (right panel). Values shown are the mean ± s.d. of three independent experiments.

To investigate the regulation of IRE1α expression by p53, we first looked at the effect of p53 on *IRE1*α mRNA expression. *IRE1α* mRNA levels were unchanged in HCT116 *p53*^+/+^ and HCT116 *p53*^−/−^ cells (Figure 3A), suggesting that p53 may play a role in post-translational regulation of IRE1α protein stability. The stability of IRE1α protein was analyzed by Western blot analysis over a time course after addition of *de novo* protein synthesis inhibitor, cycloheximide, in HCT116 *p53*^+/+^ and *HCT116 p53*^−/−^ cells. IRE1α protein was degraded in HCT116 *p53*^+/+^ cells (approximately 50% decrease at the endpoint), but the stability of IRE1α was significantly increased in HCT116 *p53*^−/−^ cells (approximately 30% decrease at the endpoint) (Figure 3B). p53 protein was also degraded by cycloheximide in a time-dependent manner and this degradation affected the speed of IRE1α protein degradation in HCT116 *p53*^+/+^ cells: after cycloheximide treatment, IRE1α protein was rapidly degraded from 0 hour to 2 hour in HCT116 *p53*^+/+^ cells, but IRE1α degradation rate was the same as HCT116 *p53*^−/−^ cells from 2 hour to 8 hour) (Figure 3B). These results suggest that IRE1α expression is regulated by p53-dependent degradation.

### The interaction of p53 with Synoviolin (SYVN1) stimulates SYVN1-IRE1α-dependent degradation of IRE1α

**Figure 4:**
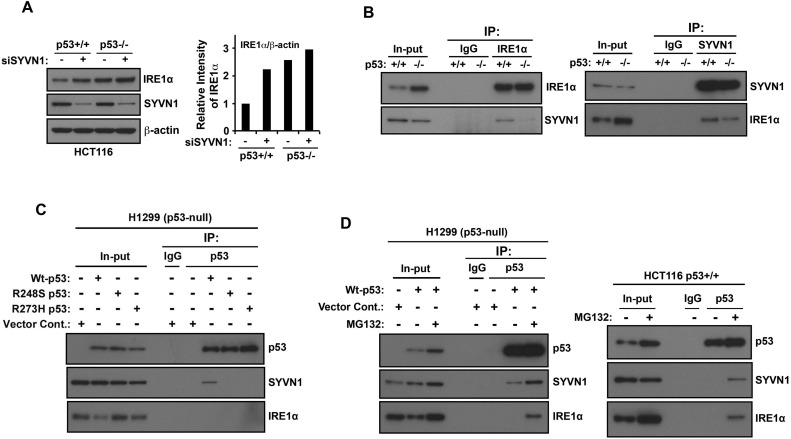
Synoviolin promotes IRE1α degradation in a wild-type p53-dependent manner **A.** SYVN1 suppresses IRE1α protein expression in wild-type p53 cells. HCT116 *p53*^+/+^ or HCT116 *p53*^−/−^ cells were transfected with siControl (−) or siSYVN1 (+) and cultured for 24 h. Cell lysates were analyzed using western blotting with indicated the antibodies (left panel). The intensities of the SYVN1 bands were quantified. The levels of SYVN1 are reported relative to those of β-actin (right panel). The blot was cut based on the size of proteins or stripped and reprobed. **B.** IRE1α and SYVN1 interaction is suppressed in p53-deficient cells. Proteins were cross-linked with DSP before protein extraction. Coimmunoprecipitation was performed with cell lysate using an IRE1α or an SYVN1 antibody. **C.** SYVN1 interacts with wild-type p53. H1299 cells transiently expressed wild-type p53, p53-R248S, or p53-R273H. Coimmunoprecipitation experiments were performed using the anti-p53 antibody. **D.** p53-SYVN1-IRE1α complex is observed by treatment with proteasome inhibitor. H1299 cells transiently expressing wild-type p53 (left panel) or HCT116 *p53*^+/+^ (right panel) cells were treated with 50 μM MG132 for 3 h. Coimmunoprecipitation experiments were performed using the anti-p53 antibody.

SYVN1 is an ER transmembrane E3 ubiquitin ligase and is directly associated with IRE1α to promote the ubiquitination and proteasomal degradation of IRE1α [16]. Therefore, we asked whether p53 regulates this process. When SYVN1 expression was inhibited by transfecting HCT116 *p53*^+/+^ cells with *SYNV1* siRNA, IRE1α expression increased (Figure 4A). In contrast, only a minor increase in IRE1α expression was observed in HCT116 *p53*^−/−^ cells transfected with *SYNV1* siRNA. Furthermore, the levels of SYVN1 were not altered by the presence or absence of p53. Next, we performed reciprocal immunoprecipitation experiments to determine whether p53 expression affects the association between SYVN1 and IRE1α in HCT116 *p53*^+/+^ and HCT116 *p53*^−/−^ cells. We detected an association between endogenous SYVN1 and IRE1α in HCT116 *p53*^+/+^ cells, and this association was suppressed in HCT116 *p53*^−/−^ cells (Figure 4B). These results suggest that p53 expression may stimulate the association between SYVN1 and IRE1α.

Next, we examined whether wild-type p53 directly interacts with SYVN1–IRE1α complex. Wild-type p53 and two p53 mutants (R249S and R273H) were overexpressed in p53-null H1299 cells and subjected to coimmunoprecipitation analysis using an anti-p53 primary antibody. The wild-type but not mutant forms of p53 immunoprecipitated with SYVN1 (Figure 4C). Furthermore, wild-type and mutant forms of p53 did not directly associate with IRE1α. Based on these results, p53–SYVN1–IRE1α complex may exist but is undetectable due to possible rapid degradation of IRE1α by the proteasome. Thus, we investigated whether p53–SYVN1–IRE1α complex could be detected by inhibition of the proteasome function. Wild-type p53 overexpressed p53-null H1299 cells or HCT116 p53^+/+^ cells were treated with the proteasome inhibitor MG132 and subjected to coimmunoprecipitation analysis using an anti-p53 primary antibody. Pretreatment with the proteasome inhibitor MG132 increased IRE1α expression and enabled the detection of the p53-SYVN1-IRE1α complex (Figure 4D). However, p53–SYVN1–IRE1α complex was not observed in the absence of MG132, indicating that this complex is likely to be disrupted by the proteasome-mediated degradation. Collectively, these results suggest that wild-type p53, SYVN1 and IRE1α form a triple complex and IRE1α is subsequently degraded by the proteasome. Thus, loss of p53 function disrupts the regulation of IRE1α by SYVN1, and as a result, protects IRE1α from proteasomal degradation and elevates IRE1α protein expression.

### The secretory function of ER is inhibited in cells expressing wild-type p53 in an IRE1α-dependent manner

**Figure 5:**
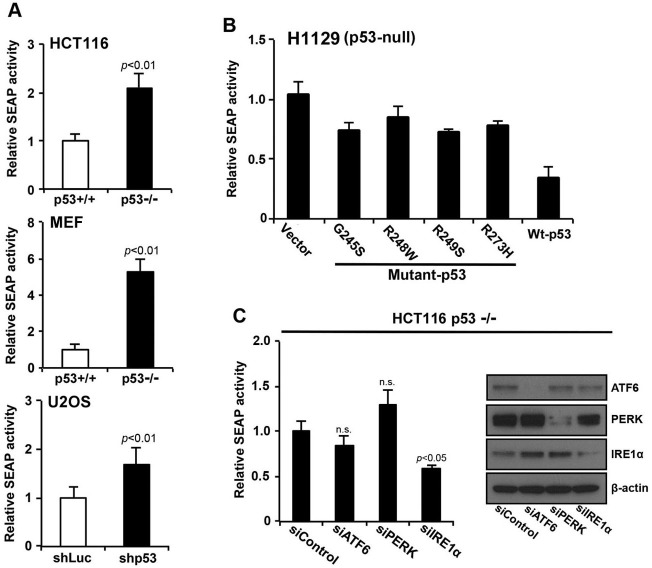
p53 deficiency increases secretory the function of the ER through the IRE1α/XBP1 pathway **A.** HCT116 *p53*^+/+^ or HCT116 *p53*^−/−^ cells, MEF *p53*^+/+^ or MEF *p53*^−/−^ cells, and U2OS shLuc or U2OS shp53 cells expressing secreted embryonic alkaline phosphatase (SEAP) were transduced with a pSEAP2 control vector and washed 24 h after transduction. The medium was then changed, and the cells were cultured for another 6 h. Culture media were analyzed for SEAP activity, and luminescence was normalized to cell number. The transfection efficiencies of HCT116 *p53*^+/+^ and HCT116 *p53*^−/−^ cells were approximately 80% each (data not shown). **B.** Overexpression of wild-type p53 inhibited SEAP activity. SEAP activities of cells that constitutively expressed the indicated p53 molecules were analyzed using the same procedure described in (A). **C.** HCT116 *p53*^−/−^ cells that expressed SEAP were transfected with siControl, siATF6, siPERK, or siIRE1α, cultured for 24 h, and following a change of medium, the cells were cultured for another 6 h. Whole cell lysates were analyzed using western blotting with the indicated antibodies, and culture supernatants were analyzed for SEAP activity. Values shown are the mean ± s.d. of three different experiments simultaneously measured. The *P* value was calculated using two-way ANOVA.

The IRE1α/XBP1 pathway is critical for the secretory function of ER [8, 18]. Since we've shown that the loss of p53 function activates the IRE1α/XBP1 pathway, we next investigated the effect of p53 on the secretory output of ER using the secreted alkaline phosphatase (SEAP) assay [19]. We expressed SEAP in HCT116 *p53*^+/+^ and HCT116 *p53*^−/−^ cells and monitored its secretion. Overexpression of SEAP induced a weak ER stress response, which was indicated by the expression of BiP and P-eIF2α (data not shown). Loss of p53 function significantly enhanced secretion of SEAP by approximately 2-fold compared with control cells (Figures 5A and S1C). Furthermore, we examined whether the increased ER function, as seen from increased SEAP activity, is due to increased ER mass and capacity. Immunofluorescence microscopy demonstrated that DsRed proteins labeled with KDEL ER localization motif were more widely distributed throughout the cytoplasm of HCT116 *p53*^−/−^ cells than the HCT116 *p53*^+/+^ cells; this is consistent with an increase in ER mass (Figure S3). Next, we examined the effect of forced expression of mutant forms (p53-G245S, p53-R248W, p53-R249S, and p53-R273H) or wild-type p53 on SEAP activity in p53-null H1299 human cancer cells. Expression of the p53 mutants inhibited secretion of SEAP to a lesser extent than that of wild-type p53 (Figure 5B). To determine if p53 contributes to ER function through the IRE1α/XBP1 pathway, secretion of SEAP in HCT116 *p53*^−/−^ cells was measured under siRNA knockdown of ATF6, PERK, or IRE1α. Inhibition of SEAP secretion was only seen in siIRE1α (Figure 5C). Together, these findings suggest that the secretory function of ER was enhanced by loss of p53 function through the IRE1α/XBP1 pathway.

### IRE1 inhibitor STF-083010 limits the growth of p53-deficient cells *in vivo*

**Figure 6:**
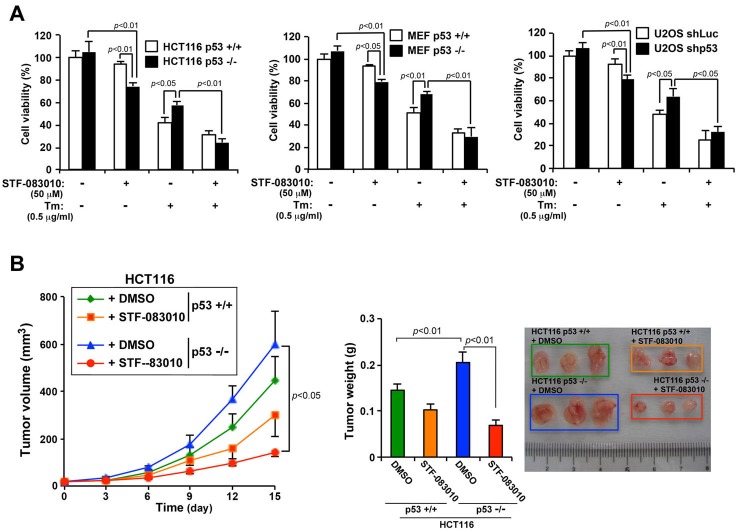
The IRE1α inhibitor (STF-083010) suppresses the growth *in vitro* and *in vivo* of p53-deficient human cancer cells **A.** Effects of an IRE1α inhibitor on cell viability and on Tm-induced cell death in p53-deficient cells. HCT116 *p53*^+/+^ or HCT116 *p53*^−/−^ cells, MEF *p53*^+/+^ or MEF *p53*^−/−^ cells, and U2OS shLuc or U2OS shp53 cells were treated with Tm (0.5 mg/mL), STF-083010 (50 μM), or both for 24 h. Cell viability was determined using an MTT assay. Values shown are the mean ± s.d. of three different experiments measured simultaneously. **B.** An IRE1α inhibitor selectively suppresses the growth of p53-deficient tumors in nude mice. HCT116 *p53*^+/+^ and HCT116 *p53*^−/−^ cells were used to engraft nude mice, and 4 days after injecting the cells, DMSO or STF-083010 (40 mg/kg) was intraperitoneally administered once every 3 days. Tumor volume was measured on the indicated days. After 15 days, the weights of the tumors (left panel) were measured. Values shown are the mean ± standard error of the mean of eight mice from each group. The *P* value was calculated using two-way ANOVA.

We observed that loss of p53 function significantly increased the secretory function of ER by activating the IRE1α/XBP1 pathway. Therefore, we hypothesized that activation of the IRE1α/XBP1 pathway by the loss of p53 function imparts an ER stress resistant phenotype to cancer cells with *p53* mutations. To test this hypothesis, we evaluated whether inhibiting the IRE1α/XBP1 pathway using the IRE1 inhibitor STF-083010 would effectively inhibit the ability of p53-deficient cancer cells to proliferate and secrete SEAP. The inhibition of IRE1α was confirmed by XBP1(S) expression (Figure S2A). Treatment with STF-083010 reduced the viability of HCT116 *p53*^−/−^ cells by approximately 20% compared with that of HCT116 *p53*^+/+^ cells (Figure 6A). Furthermore, HCT116 *p53*^−/−^ cells resisted ER stress-induced cell death compared with that of HCT116 *p53*^+/+^ cells, but simultaneous treatment with STF-083010 abolished the resistance. Similar results were obtained using MEF *p53*^+/+^ and MEF *p53*^−/−^ cells and U2OS-shLuc and U2OS-shp53 cells. To determine the effect of STF-083010 on the secretory output of ER, SEAP activity was measured in HCT116 *p53*^+/+^ and HCT116 *p53*^−/−^ cells treated with DMSO or STF-083010. SEAP activity was clearly suppressed in HCT116 *p53*^−/−^ cells treated with STF-083010 (Figure S2B), showing that inhibition of the IRE1α/XBP1 pathway abrogates the increased ER function from the loss of p53 function. Together, this suggests that activation of the IRE1α/XBP1 pathway in cancer cells with loss of p53 function promotes ER stress resistant phenotype.

To further determine whether inhibition of the IRE1α/XBP1 pathway in p53-deficient cells suppresses tumor growth, mice were engrafted with HCT116 *p53*^+/+^ and HCT116 *p53*^−/−^ cells, and the effect of STF-083010 on tumor growth was observed. Tumors induced by HCT116 *p53*^−/−^ cells aggressively grew compared with those induced by HCT116 *p53*^+/+^ cells. However, administration of STF-083010 to tumors induced by HCT116 *p53*^−/−^ cells significantly reduced tumor volume and weight by 75% and 73% at the endpoint, respectively (Figure 6B). In contrast, the respective values for STF-083010-treated HCT116 *p53*^+/+^ cells did not significantly differ (approximately 32% and 28%). Thus, the antitumor effect of STF-083010 was limited to p53-deficient tumors. Together, these data suggest that the malignant phenotype of p53-deficient tumors may be attributed to activation of the IRE1α/XBP1 pathway.

## DISCUSSION

We report here the discovery that the IRE1α/XBP1 pathway is regulated by p53. We show that loss of p53 function enhanced the secretory function of ER and suppressed ER stress-mediated cell death through activation of the IRE1α/XBP1 pathway. Furthermore, we show that the interaction between wild-type p53 and SYVN1 enhanced the association of SYVN1 and IRE1α, which is critical for proteasome-dependent degradation of IRE1α.

ER membrane protein homeostasis is controlled by ER-associated degradation (ERAD) [20]. In general, a misfolded or unfolded protein is detected by an adaptor protein, such as an ER chaperon, and is ubiquitinated by E3 ubiquitin ligase, and the ubiquitinated protein is transported from ER to the cytosol and degraded by the proteasome [20]. Recent studies report that ERAD is involved in the turnover of several proteins, but the mechanism is unclear [21, 22]. SYVN1 promotes the ubiquitination and degradation of IRE1α; however, the mechanism of regulation of the interaction between SYVN1 and IRE1α is undetermined [16]. In the present study, we discovered that p53 is a key factor that stimulates the association of SYVN1–IRE1α through its interaction with SYVN1, indicating that the p53–SYVN1–IRE1α complex effectively utilizes IRE1α as a substrate. These results provide a new insight into the regulation of homeostatic protein expression by ERAD.

The accumulating evidence clearly indicates that increasing ER function through UPR, particularly through the IRE1α/XBP1 pathway, is critical for oncogenesis by adapting tumor cells to ER stress and the secretion of growth factors [6, 10, 12]. However, the regulatory mechanism of the activation of the IRE1α/XBP1 pathway was not identified by these studies. We also showed that cancer cell lines expressing mutant forms of p53 expressed high levels of IRE1α. Furthermore, XBP1(S) expression was induced in these cells in the absence of stress. Therefore, our data suggest that p53 is a crucial regulator for ER function, and loss of p53 function induced upregulation of IRE1α expression, which increases ER function. In p53 deficient cells, while the IRE1α/XBP1 pathway was upregulated, ER stress dependent activation of ATF6 and PERK/eIF2α pathway was suppressed. These results are consistent with a previous study, where p53 deficiency suppressed the phosphorylation of eIF2α by zebularine induced ER stress [23]. It has been previously reported that overexpression of BiP suppresses PERK and ATF6 activation, subsequently becoming resistant to ER stress [24–26]. Thus, upregulation of BiP by activation of IRE1α/XBP1 pathway in the absence of stress may suppress activation of ATF6 and PERK/eIF2α pathway by ER stress in p53 deficient cells. Further ER stress transactivates proapoptotic p53-target genes, such as *PUMA* and *NOXA*, which is implicated in the induction of cell death during ER stress [27]. Thus, this pathway is involved in the loss of p53-mediated resistance to ER stress-induced cell death. Together, these data suggest that wild-type p53 suppresses ER function, and the adaptation to ER stress by downregulating IRE1α expression is a consequence of the tumor suppressor activity of p53.

An important aspect of the present study is the demonstration that an IRE1 inhibitor STF-083010 selectively inhibited the growth of tumors induced by p53-null human cancer cells in nude mice, suggesting that IRE1 inhibitors may serve as anticancer drugs that target mutant forms of p53. Cancer cells with *p53* mutations are highly malignant and aggressive, and activation of the IRE1α/XBP1 pathway contributes to this malignant phenotype [28, 29]. DNA damaging agents are widely used to treat various types of cancer, and their efficacy depends on the tumor suppressor activity of wild-type p53 [28, 30]. Thus, cancer cells that express mutant forms of p53 are resistant to numerous conventional anticancer agents. Furthermore, such cancers are characterized by more aggressive phenotypes. Our data support the conclusion that activation of the IRE1α/XBP1 pathway contributes to this phenotype in cancers that express mutant forms of p53. Therefore, inhibiting the activation of the IRE1α/XBP1 pathway may represent a promising new modality for treating cancers that lack p53 function. Taken together, our data reveal a previously unidentified mechanism mediated by p53 that maintains ER function through the regulation of the activation of the IRE1α/XBP1 pathway by SYVN1-dependent proteasomal degradation of IRE1α.

## MATERIALS AND METHODS

### Xenograft model

The Animal Research Committee of Kochi Medical School approved all experimental protocols and surgical procedures (Permit Number: H-00023). Each BALB/c nude mouse (male, 5 weeks of age) was subcutaneously inoculated in the right and left hind footpads with 5 × 10^6^ HCT116 *p53*^+/+^ or HCT116 *p53*^−/−^ cells. Four days later, DMSO or STF-083010 (40 mg/kg) was intraperitoneally administrated every 3 days. Tumors were measured every 5 days, and their volumes were calculated using the equation mm^3^ = (length (mm)) × (width (mm))^2^/2).

### Cell lines and generation of stably transfected cell lines

U2OS, H1299, HCT116 *p53*^+/+^, HCT116 *p53*^−/−^, HT1080, ME-180, A549, A172, Calu3, LS174T, MDAMB468, SK-BR-3, HCC1937, MDAMB435, MDAMB231, AU565, SUM149, WiDr, SNU1040, SW480, EJ, T24, and RD cells were maintained in DMEM supplemented with 10% FBS, 100 U/mL penicillin, and 100 μg/mL streptomycin. MEF *p53*^+/+^ and MEF *p53*^−/−^ cells were maintained in DMEM supplemented with 10% FBS, 1 % NAEE and 0.5 % *2*-mercaptoethanol. All cells were maintained at 37°C in an atmosphere containing 5% CO_2_. Wild-type p53, p53-G245S, p53-R248W, p53-R249S, p53-R273H, shp53 (pLKO.1 *p53* shRNA-753 and -814 from Sigma-Aldrich (TRCN0000003753)), and shLuc (pLKO.1 *Luciferase* shRNA Control, Sigma-Aldrich) constructs were introduced into HCT116 *p53*^+/+^, U2OS, or H1299 cells using lipofection. Cells transfected with these plasmids were selected using G418 or puromycin for 2 weeks. Experiments were performed using stable, pooled clones. HCT116 *p53*^−/−^, H1299, AU565, and SNU1040 cells were transiently transfected using lipofection with plasmids that expressed wild-type p53, p53-R249S, or p53-R273H.

### SEAP assay

Cells were transduced with the pSEAP2-Control Vector (Clontech). The cells were washed 24 h after transfection, transferred to plates containing fresh media, and then cultured for 2 h. Culture supernatants were harvested and assayed for SEAP activity using the Great EscAPe SEAP Reporter System (Clontech) [19].

### Immunoprecipitation

Cells were washed with PBS and incubated with or without PBS containing 1 mM dithiobis [succinimidyl propionate] (DSP) for 30 min, and the reaction was quenched by adding 50 mM Tris (pH 8.0) for 3 min. Cells were lysed in Tris lysis buffer (50 mM Tris-HCl (pH 7.4), 1 mM EDTA, 1 % Triton-X, 1 mM NaF, protease inhibitor mix (Nakarai), and incubated on ice for 15 min. Cellular debris was pelleted by centrifugation at 14,000 rpm for 10 min at 4°C. Primary antibody was covalently linked to protein A/G plus-agarose or protein A/G magnetic beads. Immunoprecipitated products were incubated with LDS sample buffer containing 50 mM DTT at 95°C for 10 min.

### Immunoblotting analysis

Immunoblotting experiments were conducted as previously described [31]. Antibodies used for immunoblotting were specific for the proteins as follows: BiP, P-eIF2α, eIF2α, PARP, Synoviolin1, PERK, and IRE1α (Cell Signaling); GADD34, P-PERK, XBP1, ATF6, MDM2, and p53 (Santa Cruz); and β-actin (Sigma). Antibodies were diluted to 1:1000, except for anti-β-actin (1:10000). Secondary antibodies were purchased from Promega (antirabbit and antimouse at 1:5000) or Rockland (TrueBlot antimouse at 1:1000).

### Real-time quantitative PCR and RT-PCR

Real-time quantitative PCR (qRT-PCR) and RT-PCR were conducted as previously described [31]. To normalize the amount of total RNA present in each reaction, *GAPDH* cDNA served as an internal standard. The primers used were (name: forward primer & reverse primer): *IRE1α*: 5′-TCAAACCTCATGGGTTCTCC-3′ and 5′-GTGTCATCCAACGTGGTCAG-3′; *GAPDH*: 5′-CTCAGACACCATGGGGAAGGTGA-3′ and 5′-ATGATCTTGAGGCTGTTGTCATA-3′: *p53*: 5′-AGAGTCTATAGGCCCACCCC-3′ and 5′-GCTCGACGCTAGGATCTGAC-3′. The cDNAs were used in RT-PCR to detect spliced and non-spliced forms of *XBP1* mRNA. The primers used were (name: forward primer and reverse primer): *XBP1*: 5′-GGAGTTAAGACAGCGCTTGGGGA-3′ and 5′-TGTTCTGGAGGGGTGACAACTGGG-3′.

### Immunofluorescence analysis

HCT116 *p53*^+/+^ and HCT116 *p53*^−/−^ cells were stably transfected with KDEL-DsRed2 (Clontech: pDsRed-ER vector), and treated cells were fixed in 4% paraformaldehyde for 15 min at room temperature. Cells were then extensively washed to remove any debris. All images were acquired using an Olympus confocal microscope and processed using Adobe Photoshop software.

### Cell viability assays

Cell viability was determined using the MTT method [32]. After treatment with Tm, STF-083010, or both, cells were incubated with MTT solution (1 mg/mL) for 2 h. Isopropanol and HCl were added to the final concentrations of 50% and 20 mM, respectively. The optical density at 570 nm was determined using a spectrophotometer using a reference wavelength of 630 nm.

### siRNA experiments

U2OS cells were transfected with an siRNA specific for synoviolin [33] (ON-TARGETplus siRNA, Dharmacon), ATF6, PERK, IRE1α (functionally validated siRNAs from Qiagen) and Control (Santa Cruz) [31] at final concentrations of 50 nM, using X-tremeGENE transfection reagent (Roche) according to the manufacturer's instructions.

### Statistical analysis

Differences between mean values were evaluated using two-way ANOVA followed by Tukey's test or the Student *t* test for unpaired results of more than two experimental groups. Differences were considered statistically significant for *P* < 0.05.

## SUPPLEMENTARY FIGURES AND TABLES


