# Supplementary material for: Loss of p53 enhances the function of the endoplasmic reticulum through activation of the IRE1α/XBP1 pathway
Source: Oncotarget. 2015 Jun 23;6(24):19990–20001. doi: 10.18632/oncotarget.4598 (PMC4652982; doi:10.18632/oncotarget.4598)
Supplement: Supplementary file 1 [file oncotarget-06-19990-s001.pdf]

Loss of p53 enhances the function of the endoplasmic reticulum through activation of the IRE1α/XBP1 pathway

Supplementary Material

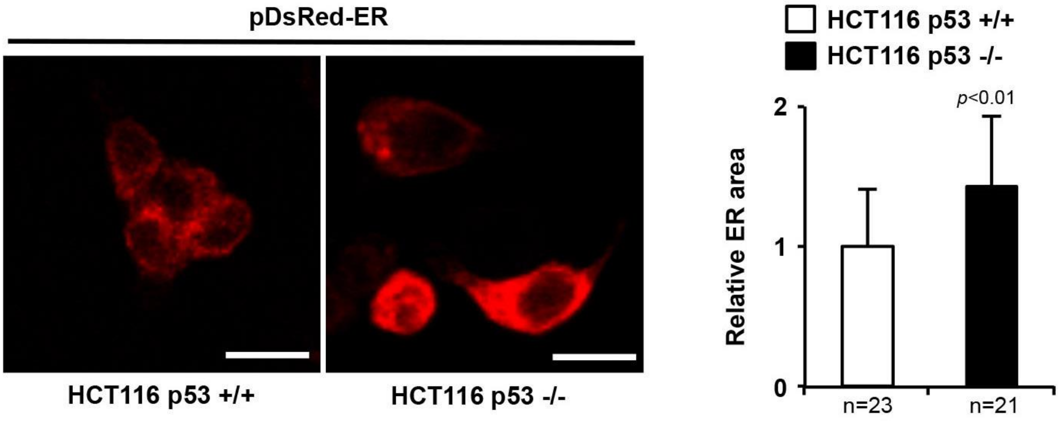

Supplementary Fig. S1. The amount of the ER is increased in p53-deficient cells. HCT116 *p53*$^{+/+}$ and HCT116 *p53*$^{-/-}$ cells expressing calreticulin-RFP were transduced with the pDsRed-ER vector to visualize the amount of ER using confocal microscopy. Right panel: The area of the ER area was calculated using Image J software (NIH) in three different fields (HCT116 *p53*$^{+/+}$, 23 cells; HCT116 *p53*$^{-/-}$, 21 cells). Values shown are the mean ± s.d. The *P* value was calculated using two-way ANOVA.

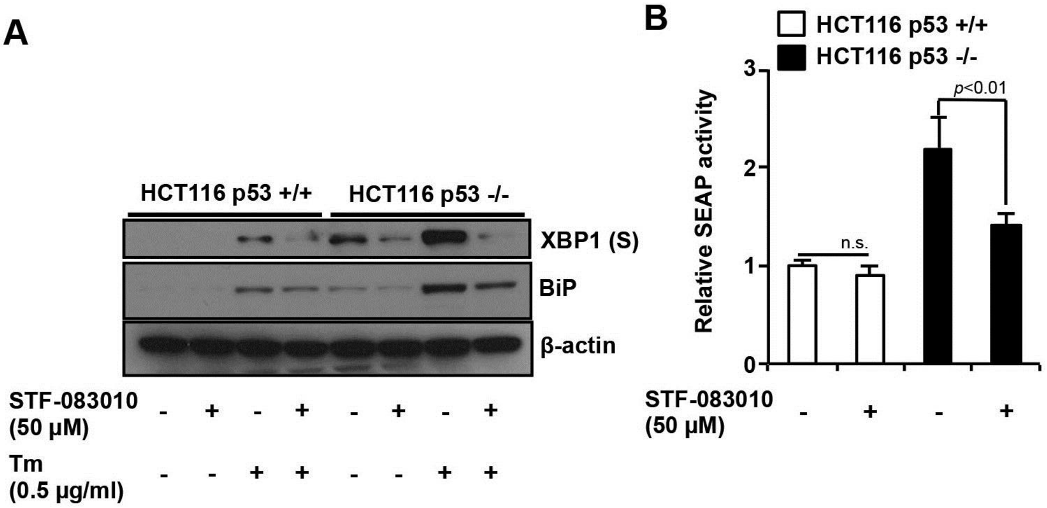

Supplementary Figure S2. An IRE1α inhibitor inhibits the secretory function of the ER in p53-deficient cells. (A) HCT116 *p53*$^{+/+}$ or HCT116 *p53*$^{-/-}$ cells were treated with Tm (0.5 mg/mL), STF-083010 (50 μM), or both for 6 h. Whole cell lysates were analyzed using western blotting with the indicated antibodies. (B) An IRE1α inhibitor inhibits the secretory function of the ER in p53-deficient cells. HCT116 *p53*$^{+/+}$ and HCT116 *p53*$^{-/-}$ cells that expressed SEAP were treated with DMSO (−) or STF-083010 (50 μM) (+) for 12 h. SEAP assays were performed using the same procedure described in Figure 4.
